# Modification of Polyvinyl Chloride Composites for Radiographic Detection of Polyvinyl Chloride Retained Surgical Items

**DOI:** 10.3390/polym15030587

**Published:** 2023-01-23

**Authors:** Martina Polaskova, Tomas Sedlacek, Zdenek Polasek, Petr Filip

**Affiliations:** 1 Centre of Polymer Systems, Tomas Bata University in Zlín, Trida Tomase Bati 5678, 760 01 Zlín, Czech Republic; 2 Department of Food Technology, Faculty of Technology, Tomas Bata University in Zlín, Vavreckova 275, 760 01 Zlín, Czech Republic; 3 Institute of Hydrodynamics, Czech Academy of Sciences, Pod Patankou 5, 166 12 Prague, Czech Republic

**Keywords:** retained surgical items, polyvinyl chloride, bismuth oxychloride, barium sulfate, radiopacity

## Abstract

The ever-present risk of surgical items being retained represents a real medical peril for the patient and potential liability issues for medical staff. Radiofrequency scanning technology is a very good means to substantially reduce such accidents. Radiolucent medical-grade polyvinyl chloride (PVC) used for the production of medical items is filled with radiopaque agents to enable X-ray visibility. The present study proves the suitability of bismuth oxychloride (BiOCl) and documents its advantages over the classical radiopaque agent barium sulfate (BaSO_4_). An addition of BiOCl exhibits excellent chemical and physical stability (no leaching, thermo-mechanical properties) and good dispersibility within the PVC matrix. As documented, using half the quantity of BiOCl compared to BaSO_4_ will provide a very good result. The conclusions are based on the methods of rotational rheometry, scanning electron microscopy, dynamic mechanical analysis, atomic absorption spectroscopy, and the verification of zero leaching of BiOCl out of a PVC matrix. X-ray images of the studied materials are presented, and an optimal concentration of BiOCl is evaluated.

## 1. Introduction

Unintentionally retained surgical items (RSI) or retained foreign objects (RFO) represent an everlasting problem where the impact—apart from non-negligible financial costs—is significant for both medical staffs and is a danger to patients’ lives. The true RSI incidence is unknown as some patients will be initially unaware of hosting such items. The data introduced in the literature do not differ significantly: from 1 case in 1000–1500 abdominal cavity operations [1,2] to 1 case in 2000 operations under general anesthesia [3]. It should be pointed out that these data are relatively new. 

The thorough analysis presented in the literature [3,4,5,6,7] documents that the human factor that participates in a standard counting protocol increasing vigilance and communication among staff [3,8] cannot fully eliminate hard RSI events. It implies that the classical methods (standard counting, presence of a trainee—however, reducing an occurrence of RSI by two-thirds [6]) cannot prevent these adverse patient outcomes [9]. It substantiates that a parallel implementation of more sophisticated methods would be required.

Radiofrequency scanning technology represents a very efficient method for the non-invasive identification of radiopaque items [5,7,8,9,10]. The problem is that not all medical items are able to absorb the radiant energy of an electromagnetic field due to their radiolucency (electromagnetic radiation passes through such particular materials) caused by their low electron density. Such items include sponges, swabs, textile materials, and plastic items [1,4,8]. In principle, there are two possibilities of how to adapt radiolucent materials for their detection by radio-frequency identification. The first is to use tags attached to the materials (e.g., textile materials), which ensures their tracking by changes in an electromagnetic field. The alternative to this external method is the application of so-called radiopaque agents within medical plastic items. The composition of these agents is frequently based on iodine, barium, bismuth, gold, and tungsten [11,12,13,14]. The agents -quite often in the form of nanoparticles- can be either mixed with the polymer during the manufacturing process at a specific ratio or infused into the manufactured polymer by organic solvent treatment [13,15].

The radiopacity of such composite materials is then subject to the thickness of the final product and the concentration and nature of radiopaque materials used. Radiopaque materials must be chemically and physically stable during processing and under physiological conditions within the human body. In addition, factors such as particle morphology (shape and size), concentration, degree of dispersion, and interface effects between the particles and surrounding polymeric matrix can significantly influence the processability of a compound and the final properties of the composite [10,16,17,18,19,20]. High-loading filler fractions up to 60 wt.% are often required to achieve good X-ray visibility, although such amounts of radiopaque filler can strongly influence mechanical properties [21]. Therefore, the type and quantity of radiopaque material are subject to the specific properties of the individual medical devices.

Barium sulfate (BaSO_4_) represents the most widely used radiopaque agent [13,22,23,24,25,26]. Nevertheless, parallel to radiopaque efficiency, it is also necessary to analyze other aspects, such as detrimental effects on the mechanical properties of the device or biological reactions [27,28]. As a consequence, other radiopaque agents are intensively studied. Based on atomic number and handling risk, nine potential filler compounds have recently been evaluated [29]. With the development of the radiographical techniques [30], the choice of potential radiopaque candidates is gradually enlarging. In the case of polyurethanes as a basic material, radiopacity was introduced by incorporating an iodinated chain extender resulting in a favorable replacement of barium sulfate [31]. The agents based on bismuth [24,32] also proved to be very competitive. Specifically, greater attention is paid to bismuth oxychloride (BiOCl) [20,29]. Bismuth oxychloride exhibits twice the density resulting in a doubled level of radiopacity using the same filler loading as barium sulfate [33,34,35]. This significantly contributes to the maintenance of sufficient mechanical properties of a carrier polymer, ensuring no risk of overloading (causing mechanical properties deterioration) and surface smoothness. This reflects in the easier insertion of medical aids and a reduction in the likelihood of thrombus formation [16].

From the above, it is obvious that the overall characteristics of radiopaque materials (the degree of radiodensity/radiopacity, the mechanical properties, and the patient´s comfort) are subject to the specific combination of such radiolucent materials and radiopaque agents, and as such cannot be generalized. The goal of this contribution is to demonstrate the advantage of bismuth oxychloride as a radiopaque agent over classical barium sulfate for frequently used medical items made of medical-grade polyvinyl chloride (PVC). As far as the authors are aware, this compounded material is yet to be studied. Among other things, this substitution substantially helps to improve the thermo-mechanical properties of the resulting material, and better adhesion between bismuth oxychloride and the PVC matrix is exhibited when compared to barium sulfate. To this aim, radiographic, rheological, thermo-mechanical, and morphological characteristics will be determined, including verification of zero leaching of BiOCl out of a PVC matrix.

## 2. Materials and Methods

### 2.1. Materials

**Figure 1:**
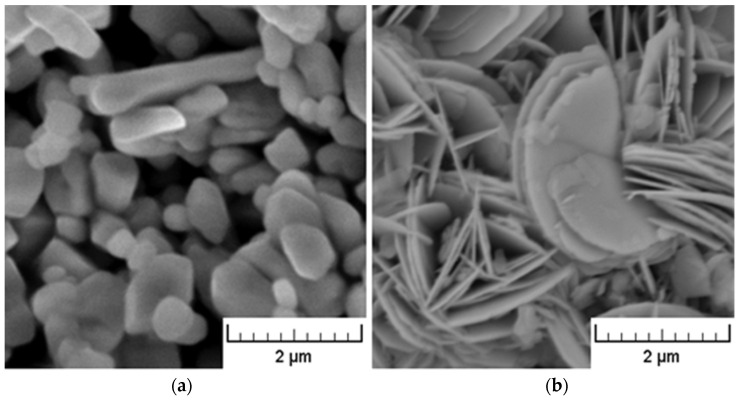
SEM pictures of BaSO_4_ powder (**a**) and BiOCl powder (**b**).

In the experiments, the medical grade polyvinyl chloride RB 3 (PVC) produced by Modenplast Medical S.R.L. (Fiorano Modenese, Italy) with a density of 1.23 g/cm^3^ (test method ISO 1183), hardness Shore A 75 (test method ISO 868), was used. Bismuth oxychloride was supplied by Sigma-Aldrich (Prague, Czech Republic) with a density of 7.70 g/cm^3^. It exhibits approximately platelet structure (see Figure 1), ensuring a smooth and shiny surface of composites. Barium sulfate powder provided by Sigma-Aldrich (Prague, Czech Republic) with a density of 4.50 g/cm^3^ exhibits–in comparison with bismuth oxychloride- a rather grainy structure (see Figure 1). Prior to mixing with the polymer matrix, it was necessary to dry BaSO_4_ powder for 15 h at a temperature of 80 °C.

### 2.2. Preparation of Compounded Samples

**Figure 2:**
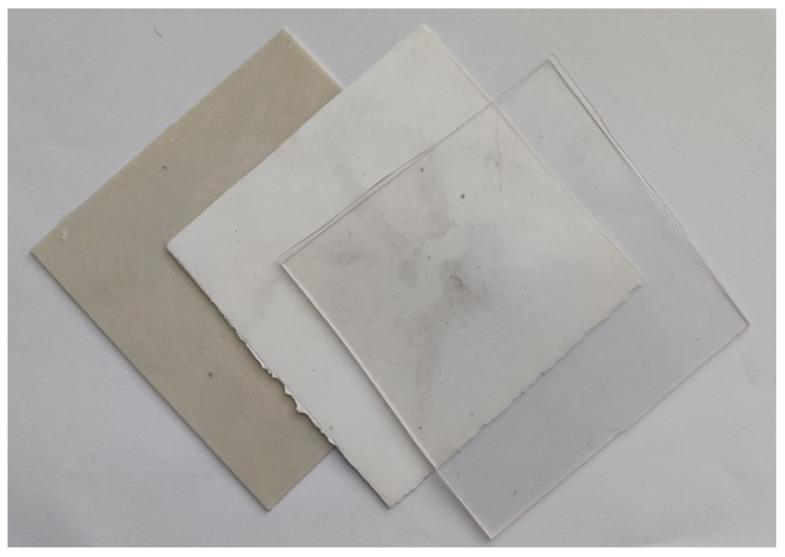
SEM pictures of the compounded material (from left: 34Ba, 20Bi, pure PVC).

PVC pellets were melt-mixed with various amounts of additives, as summarised in Table 1. Since the density of the material is directly linked to its absorption ability of X-ray photons, expressed through an attenuation coefficient, the comparative concentration ratio of the chosen fillers of 1:1.7 was derived from the different densities of BiOCl and BaSO_4_ powders. Melt-mixing process was performed via a MiniLab II Micro Compounder (ThermoHaake, Karlsruhe, Germany) at a temperature of 160 °C and screw speed rotation of 35 rpm. The compounding time of 8 min was sufficient for all compounded samples to achieve constant torque of the drive motor. Pure PVC resin was treated in the same way to ensure the identical temperature and shear history for all samples. Finally, the prepared polymer compounds and processed PVC were consequently compressions molded at a temperature of 160 °C into 0.1 and 1.0 mm thick sheets that were further employed for the preparation of testing samples. The area of these sheets was 125 mm × 125 mm. The prepared compounded material is shown in Figure 2.

### 2.3. Rheometrical Measurements

The temperature-dependent linear viscoelastic properties of the prepared compounds were determined using a rotational rheometer Gemini II (Malvern Panalytical, Malvern, U.K.) with 25 mm parallel-plate geometry and oscillatory mode in the frequency range from 0.015 to 15 s^−1^ and at a temperature of 160 °C. The flow behaviour of the tested samples of 1 mm thickness was then evaluated from the values of complex viscosity as a function of angular frequency. Each measurement (also for the other subsections) was carried out in triplicate and exhibited negligible deviations.

### 2.4. Scanning Electron Microscopy

A scanning electron microscope (SEM) Vega II LMU (Tescan, Brno, Czech Republic) was utilized to provide images of both the cross-section morphology of the prepared composites and the pristine powders. Composite samples were broken in liquid nitrogen and sputtered coated with a palladium gold alloy prior to the SEM experiments. Imaging was carried out at an accelerating voltage of 10.0 kV.

### 2.5. Dynamic Mechanical Analysis

Dynamic mechanical analysis (DMA) as a versatile thermal method was performed to obtain both the storage modulus and glass transition temperature of the prepared samples. While the storage modulus as a function of temperature provides information about the material’s thermal stability, the glass transition temperature represents the boundary between polymers’ elastic and viscoelastic behavior. Specimens with an overall diameter of 2.5 mm and thickness of 1 mm were placed in a DMA instrument—DMA/SDTA 861e (Mettler Toledo GmbH, Greifensee, Switzerland)—and oscillated at a frequency of 10 Hz in shear mode. The specimens were heated at a rate of 3 °C/min in the temperature region from −65 to 80 °C. The linear viscoelastic region of radiopaque composites was determined, prior to the testing regime, by means of a dynamic deformation sweep test at a fixed frequency of 10 Hz and temperature of −65 °C. At this temperature, the linear region of mechanical response was determined for the displacement amplitude up to 0.5 μm.

### 2.6. Atomic Absorption Spectroscopy

The stability of BiOCl filler within the PVC matrix under a physiological condition of gastric environment was evaluated with the help of atomic absorption spectroscopy. For these purposes, hydrochloric acid (HCl) of pH 2, simulating the gastric juice, was used as a leaching media. Samples of 20 mm × 20 mm × 0.1 mm were immersed into 20 mL of HCl and kept in sealed glass bottles for 7 and 30 days in a shaking incubator Cole-Parmer Stuart Orbital SI500 (Cole-Parmer Instrument Co., Ltd., St. Neots, UK) at a temperature of 37 °C and circular motion speed of 80 rpm. After chosen time periods (7 and 30 days), the samples were removed, and the presence of bismuth within the leachates was examined with the help of an atomic absorption spectrometer GBC 933 AA (GBC Scientific Equipment Pty Ltd., Braeside, Australia).

### 2.7. X-ray Measurements

Radiopaque properties of PVC composites filled with BiOCl and BaSO_4_ were recorded using a high-frequency X-ray device Gierth HF 100 Plus (Gierth X-ray International GmbH, Riesa, Germany). X-ray imaging was performed at conditions equal to a tube voltage of 70 kV, an electric charge of 1.2 mAs, and an exposure time of 0.05 s, which are within the X-ray energy range of 20–100 keV commonly used for diagnostic radiology.

## 3. Results and Discussion

### 3.1. Rheological Characterization

**Figure 3:**
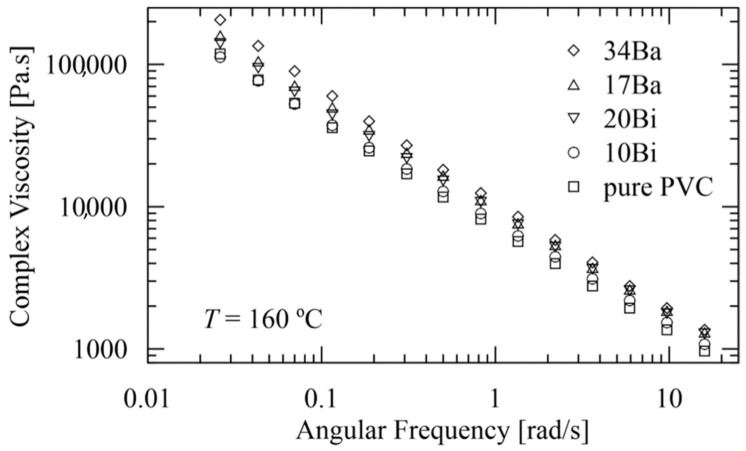
Complex viscosities of the composites and pure PVC matrix as a function of angular frequency at a temperature of 160 °C.

The complex viscosity data of the studied polymeric systems as a function of angular frequency at a temperature of 160 °C are presented in Figure 3. 

The complex viscosity for the individual composites and pure PVC matrix can be approximated by the relation:

log(*η*) = a − 0.74 × log(*ω*) (1)

where *η* [Pa.s] is the complex viscosity, and *ω* [rad/s] represents the angular frequency. The slope of all straight lines is identical (−0.74), and only the additive term a [10^a^ has units Pa.s^1.74^.rad^−0.74^] (increasing with increasing contents of particular filler) diversifies between the individual composites (see Table 2). The mean deviation of the approximated values log(*η*) using Equation (1) attains 0.4%, which—for the real non-transformed values of the complex viscosity *η*—corresponds to 5% for *ω* = 0.026 rad/s and gradually decreases to 3% for *ω* = 16 rad/s. These deviations are within the experimental errors. Equation (1), in combination with Table 2, can be used for an estimate of the behavior of the composites with different percentage participation of BaSO_4_ or BiOCl.

Generally, addition of inorganic particulate fillers into the polymer matrix results in an increase in viscosity due to the two factors. First, the mobility of macromolecules is more restricted, and second, with an increase in filler concentration, the polymer-particle interaction becomes stronger, resulting in a further increase in viscosity [18]. While the flow, permanent competition between the creation and destruction of polymer-particle interaction takes place. While at low shear rates, interactions could be easily restored, leading to higher viscosity values, at higher shear rates, destruction of polymer-particle interaction predominates, which results in minor viscosity variation [19], as documented in Figure 2. Note that in accordance with the expectation [18], the higher radiopaque filler loadings of BaSO_4_ (5.3 vol.% for sample 17Ba and 12.3 vol.% for sample 34Ba) were bounded to more significant viscosity increase compared to the flow behavior of BiOCl/PVC compounds with a filler content of 1.7 and 3.8 vol.% for samples 10Bi and 20Bi, respectively. However, these findings are not in compliance with the results of McNally and Rudy [16] and Godinho et al. [22], concluding that the incorporation of BaSO_4_ particles into the PVC matrix leads to a compound’s viscosity decrease. Since the pure PVC resin was not pre-processed in the mentioned studies, the possible explanation of revealed inconsonance can be caused by variation in the temperature history of pure PVC resin and BaSO_4_/PVC samples due to divergence in their preparation.

The graphs depicting load vs. time for PVC with the highest filler loading 34Ba and for pure PVC are introduced in the Supplement (Figure S1). As apparent, after an initial increase, the torque values are stabilized after approximately 7 min. The value corresponding to pure PVC is higher by approximately one-eighth of that for 34Ba. 

### 3.2. SEM Analysis

**Figure 4:**
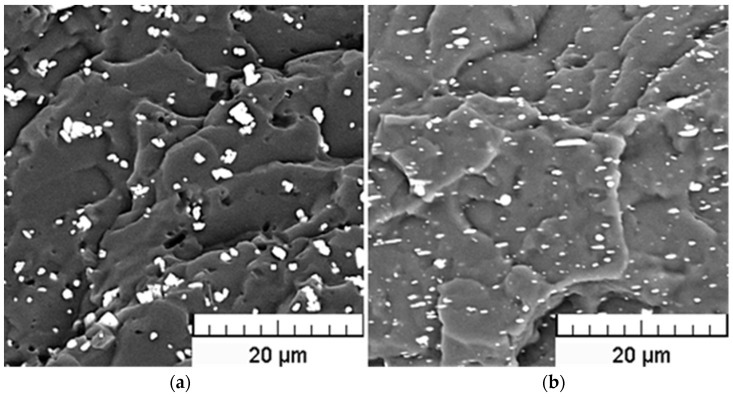
SEM pictures of the cross-sections of 17Ba (**a**) and 10Bi (**b**) samples.

**Figure 5:**
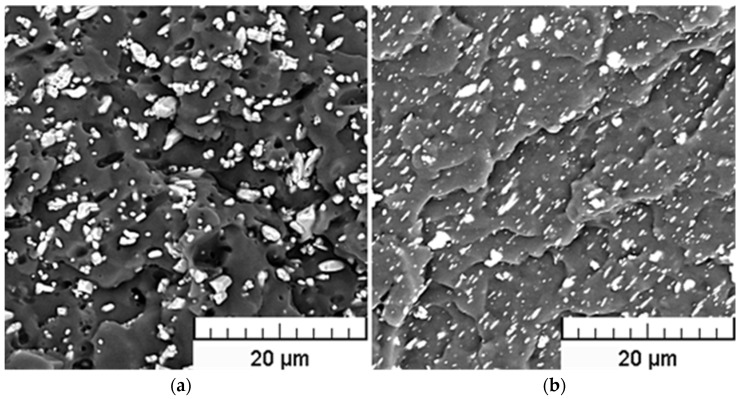
SEM pictures of the cross-sections of 34Ba (**a**) and 20Bi (**b**) samples.

Since a sufficient degree of dispersion of filler particles within the polymeric matrix is one of the factors significantly influencing the extent of polymer-particle interactions and, as such, closely related to compounding process efficiency, the resulting structures of the prepared compounds were evaluated using Scanning Electron Microscopy (SEM) technique. As obvious from Figure 4 and Figure 5, relatively uniform dispersion of the fillers within the PVC matrix was achieved for all tested filler loadings, in spite of a partial agglomeration tendency of both filler types. However, the average size of agglomerates does not exceed 5 μm.

**Figure 6:**
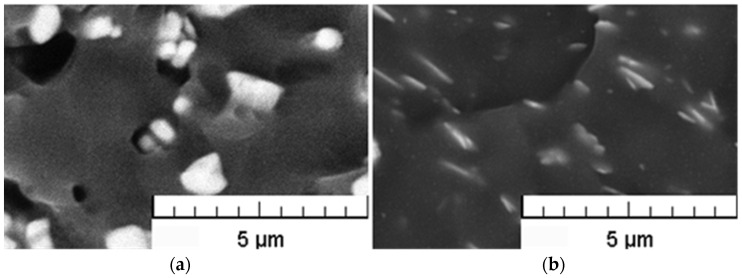
SEM details of the cross-sections of BaSO_4_/PVC (**a**) and BiOCl/PVC (**b**) samples.

The SEM details of the cross-section areas of BiOCl/PVC and BaSO_4_/PVC composites are compared in Figure 6. While bulky types of agglomerated particles are seldom found in the BaSO4/PVC samples, an apparent tendency for delamination of originally layered BiOCl particles into nanosheets (see Figure 1) can be observed in BiOCl/PVC materials. Moreover, due to its hydrophobic behavior [12], BiOCl powder is readily dispersed in the polymeric matrix, and BiOCl particles (nanosheets) are completely embedded within the PVC matrix (Figure 6 right). On the other hand, the SEM details of BaSO_4_/PVC composites revealed rather poor adhesion of BaSO_4_ filler to the PVC matrix (Figure 6 left). It is apparent that the filler particles are not wetted by the polymeric matrix. Moreover, in the monitored cross-sectional area there can be observed pores or holes probably formed during the breaking of the samples in liquid nitrogen as some particles were pulled out of the polymeric matrix.

### 3.3. Impact of Radiopaque Fillers on Thermo-Mechanical Properties

**Figure 7:**
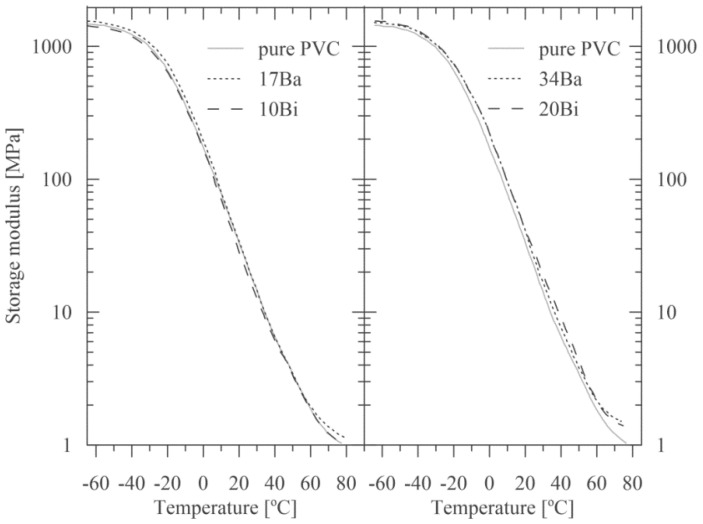
The storage modulus of the samples as a function of temperature (the semi-log coordinates).

**Figure 8:**
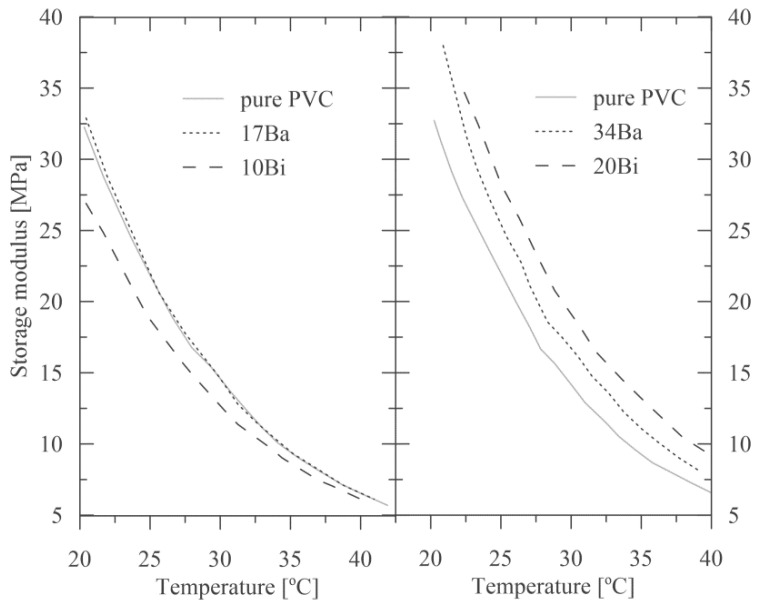
The storage modulus of the samples as a function of temperature—a detail in the normal coordinates.

Addition of the radiopaque filler into a polymeric matrix also contributes to the resulting thermo-mechanical properties of the compounds. Since mineral filler has a substantially higher modulus than polymers, its addition usually reflects in a higher modulus of polymer composite [18]. The effect of various amounts of both types of fillers on storage modulus, indicating a change of material elasticity, is presented in Figure 7 in more detail, using the normal coordinates in Figure 8. A comparison of viscoelastic behavior for pure PVC and all four compounds are presented in the Supplement (elastic and storage moduli in Figure S2, elastic and storage moduli in more detail in Figures S3 and S4, and courses of phase angles in Figure S5).

As apparent for lower temperatures, storage moduli for the individual composites are nearly identical to that of pure PVC. However, for temperatures exceeding zero, there is an increase in storage moduli for the samples 17Ba, 34Ba, and 20Bi in comparison to pure PVC (see Figure 7 and Figure 8). On the other hand, the addition of 10 wt. % of BiOCl (sample 10Bi) leads rather to a storage modulus decrease compared to unfilled PVC (see Figure 7). These results are very favorable as they prove no significant reinforcing of the polymer matrix with the addition of radiopaque fillers.

Glass transition temperature (*T*_g_) is commonly defined as the maximum damping ratio (tan *δ*) or the maximum loss modulus. Its value can also be derived from the onset of the change in the slope of the storage modulus curve. In this work, *T*_g_ was evaluated from a maximum of damping ratios of the prepared composites, as summarised in Table 3. The results document a slight *T*_g_ decrease in the samples 17Ba, 34Ba, and 10Bi compared to pure PVC. This course indicates that the radiopaque fillers incorporated into the PVC matrix -at least up to a certain concentration- can work as plasticizers by embedding themselves between the chains of polymers and spacing them apart, allowing thus a higher degree of molecular chain mobility due to reduced chain cooperativity [20]. Nevertheless, it should be noted that the addition of 20 wt. % of BiOCl (sample 20Bi) is assigned with the recovery of pristine *T*_g_ and tan *δ* values (that of PVC), indicating an improvement of adhesion at the filler-polymer interface [36].

### 3.4. Non-Toxicity of BiOCl Compounds

As BiOCl dissolves in concentrated hydrochloric acid, the stability of BiOCl/PVC systems under physiological conditions of a gastric environment [37,38], pretended by HCl of pH 2, was tested. Generally, the solid BiOCl readily dissolves in concentrated hydrochloric acid to give a clear solution of bismuth trichloride. This reaction is reversible, meaning that solid BiOCl and bismuth trichloride in the solution can exist in equilibrium.

Atomic absorption spectrometry, as an adequate method for the determination of bismuth in trace amounts [39,40], was employed. For this purpose, the standard bismuth solution (1 mg/mL) was diluted with nitric acid (65 vol.%) to prepare the calibration curves for 20, 40, and 60 ng/mL. As no peak response was registered during the measurement, it can be concluded that the potential amount of Bi leached out from BiOCl/PVC composites into HCl leaching media is below the lowest limit of the calibration curves, hence lower than 20 ng/mL.

It can be explained either by the proper embedding of BiOCl particles within the PVC matrix, to an extent preventing BiOCl particles from direct contact with leaching media, or that the pH level in the gastric environment is not low enough to dissolve BiOCl particles. By virtue of this analysis, the stability of the BiOCl/PVC system in HCl leaching media for the duration of 30 days was proven, and from this point of view, the utilization of this material, for instance, in the form of catheters can be considered as safe. This is an analogy to the compound poly(glycerol sebacate) acrylate/ bismuth oxychloride, as studied in [24]. 

In the case of the composites containing BaSO_4_, there is no leaching out of the polymeric matrix, reasoning the long tradition of BaSO_4_ utilization in medical applications. Moreover, BaSO_4_ is usually used as so-called “barium meal” as a means of imaging the digestive tract because, in this case, no toxic side products originate inside the body environment [23].

### 3.5. Radiopaque Detection

**Figure 9:**
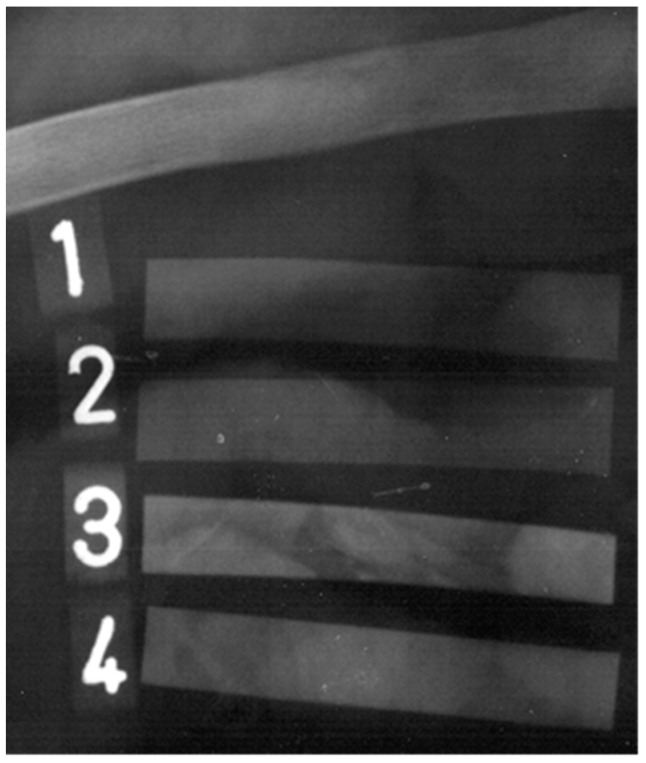
X-ray image of the radiopaque samples. The numbers 1, 2, 3, and 4 stand for the samples 10Bi, 17Ba, 20Bi, and 34Ba, respectively. In contrast, a bone is presented in the top part (above sample 10Bi).

When radiopaque material is inserted into a human body, a light-dark contrast appears on X-ray images, similar to in Figure 9. A lighter area in this picture corresponds to a smaller number of transmitted photons, while a darker area represents the transmission of a larger number of photons. This contrast is essential for an accurate location of medical aids during critical procedures inside the human body. Hence, during the final inspection and after the surgical procedure, erroneously retained aids made of radiopaque material are detectable with X-ray images. Their location is indicated by the lighter shades. Due to the different shades and the radiologist´s experience, they cannot be confused with individual bones. For comparison, an X-ray image of a bone is presented in the top part of Figure 9.

Figure 9 shows the prepared samples Ba and Bi with thicknesses of 1 mm and approximate dimensions of 10 mm × 60 mm (manually cut out from pressed sheets, the size was chosen in accordance with the size of a bone used) can be seen on the X-ray images and can be compared. It should be emphasized that BaSO_4_/PVC samples 17Ba and 34Ba (labeled as 2 and 4, respectively) were filled with almost two times higher concentrations of the filler compared to that of BiOCl/PVC samples 10Bi and 20Bi (1 and 3, respectively), rending thus comparable ability to block the passage of X-rays. Indeed, the samples with higher filler loading, 34Ba, and 20Bi, exhibit a mutually similar contrast, which is -compared to the samples 17Ba and 10Bi—also much brighter and sharper. In addition, the radiopaque properties of prepared samples can be confronted with the contrast of a bone, which is captured in the top part. The question is, which is the optimal concentration for a radiopaque agent. The dominant criterion should be good visibility of erroneously retained aids and the minimum failure in their recognition, as it is still necessary to keep in mind that the evaluation of X-ray images is based on the human factor. In this regard, the dimensions of the aids, and especially the thickness of the employed radiopaque layer, can be even lower than 1 mm, which is the thickness of the samples presented in Figure 9.

On the other hand, there is financial availability of radiopaque materials. With regards to acquisition costs of individual radiopaque agents, according to a pricelist of Sigma Aldrich (Prague, Czech Rep.), the price of BiOCl has more than doubled compared with BaSO_4_. However, as shown, only half the quantity of BiOCl is sufficient. The remaining difference is more than adequately balanced by the better properties of the BiOCl-PVC compound: thermo-mechanical parameters, adhesion, and stability. Based on the necessary contents of BiOCl and the overall superior quality of the proposed compound, the price of this radiopaque material is viable.

## 4. Conclusions

At present, the radiopacity of medical items used within the human body should be an inevitable attribute serving to identify their location and to substantially reduce these unintentionally retained surgical items. Radiopaque agents should not degrade the properties of the basic materials used, such as their thermo-mechanical characteristics and, crucially, their biocompatibility and non-toxic properties. The emphasis should be paid to a limited content of radiopaque agents, however, ensuring their errorless detection. 

Bismuth oxychloride proved to be a very good candidate for fulfilling all these demands. Very good biocompatibility and negligible in vivo toxicity are discussed in [41,42]. Further, BiOCl is a water-insensitive material that exhibits extremely low toxicity as a result of its almost complete insolubility in aqueous solutions such as biological fluids [43]. Favorable results concerning thermo-mechanical characteristics are presented in Section 3.3. It was shown that BiOCl exhibits comparable properties to the frequently used barium sulfate, but for the case of the PVC matrix, BiOCl weight can be reduced by nearly one-half in comparison with barium sulfate. It has a favorable impact, for instance, on the thermo-mechanical properties of the composite. Moreover, a better adhesion between bismuth oxychloride and polymeric matrix was observed compared to barium sulfate.

## Figures and Tables

**Table 1:** Composition of prepared samples.

Sample Label	BaSO_4_ (*ρ* = 4.5 g/cm^3^)		BiOCl (*ρ* = 7.7 g/cm^3^)	
	wt.%	vol.%	wt.%	vol.%
Pure PVC	-	-	-	-
17Ba	17	5.3	-	-
34Ba	34	12.3	-	-
10Bi	-	-	10	1.7
20Bi	-	-	20	3.8

**Table 2:** The values of an additive constant as for the individual composites and pure PVC matrix.

Material	Notation	Parameter a
Pure PVC matrix	PVC	3.86
PVC matrix + 17 wt.% BaSO_4_	17Ba	3.99
PVC matrix + 34 wt.% BaSO_4_	34Ba	4.03
PVC matrix + 10 wt.% BiOCl	10Bi	3.88
PVC matrix + 20 wt.% BiOCl	20Bi	3.96

**Table 3:** Effect of radiopaque fillers on the phase transition behavior.

Material	*T*_g_ [°C]	tan *δ*
Pure PVC	24	0.42
17Ba	22	0.46
34Ba	23	0.46
10Bi	22	0.46
20Bi	24	0.42

## Supplementary Materials

The following supporting information can be downloaded at: https://www.mdpi.com/article/10.3390/polym15030587/s1, Figure S1: Load in dependence on time for pure PVC and the highest filler loading 34Ba; Figure S2: A comparison of viscoelastic behaviour of pure PVC and all four compounds; Figure S3: A comparison of elastic moduli of pure PVC and all four compounds; Figure S4: A comparison of storage moduli of pure PVC and all four compounds; Figure S5: A comparison of phase angles tan δ of pure PVC and all four compounds.
