# Supplementary material for: Modification of Polyvinyl Chloride Composites for Radiographic Detection of Polyvinyl Chloride Retained Surgical Items
Source: Polymers (Basel). 2023 Jan 23;15(3):587. doi: 10.3390/polym15030587 (PMC9919178; doi:10.3390/polym15030587)
Supplement: Supplementary file 1 [file polymers-15-00587-s001.zip › polymers-2131650-supplementary.pdf]

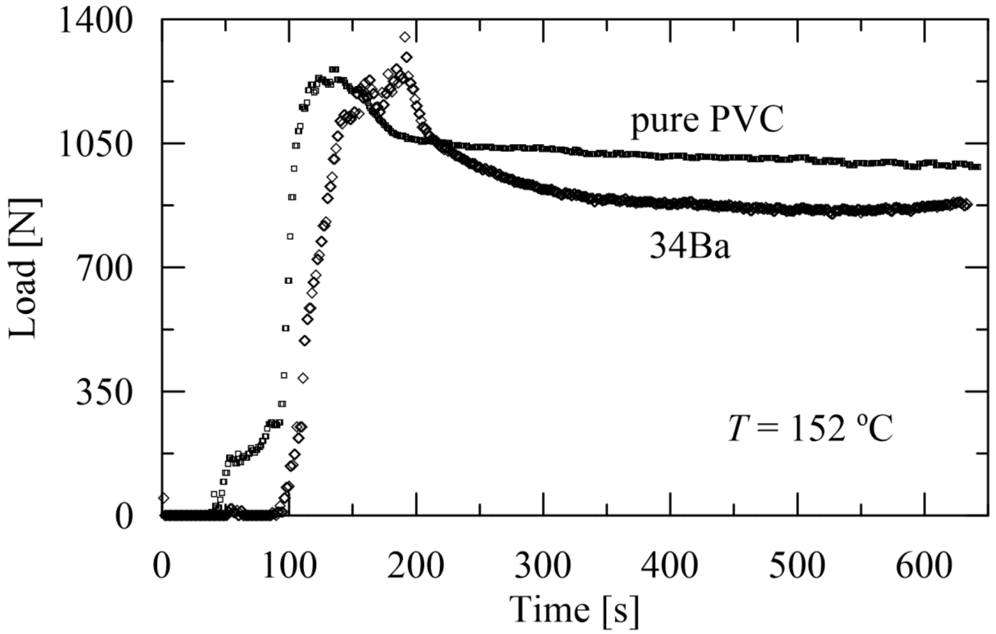

Figure S1. Load in dependence on time for pure PVC and the highest filler loading 34Ba.

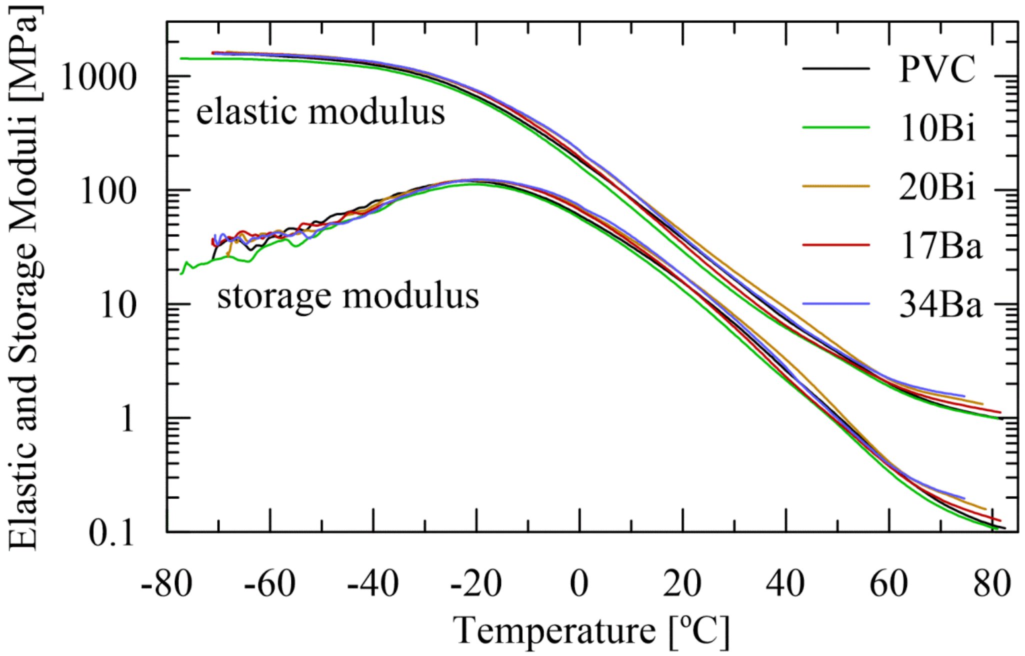

Figure S2. A comparison of viscoelastic behaviour of pure PVC and all four compounds.

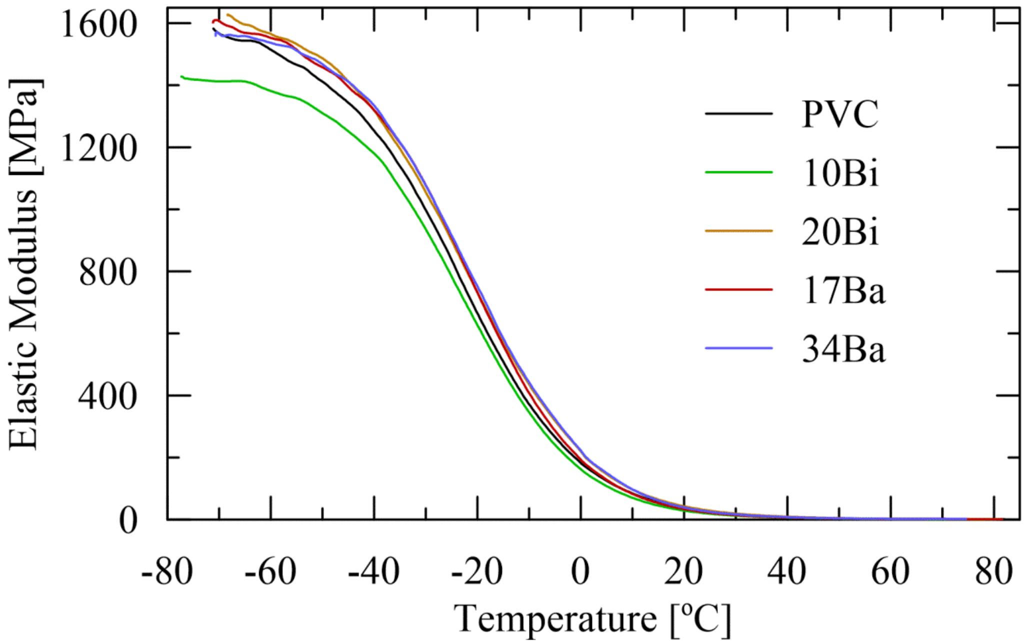

Figure S3. A comparison of elastic moduli of pure PVC and all four compounds.

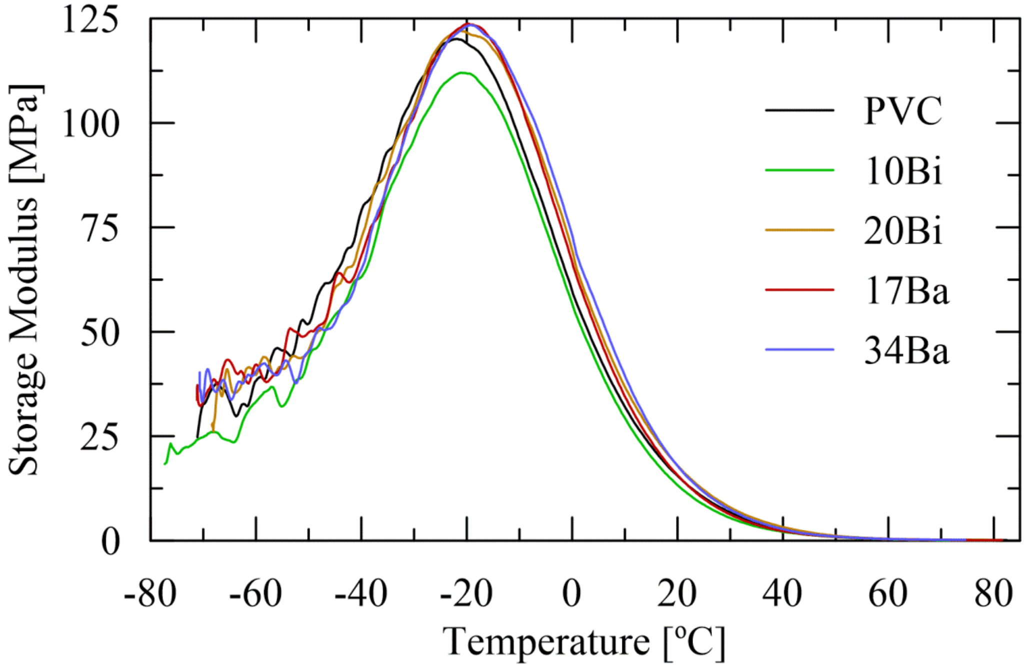

Figure S4. A comparison of storage moduli of pure PVC and all four compounds.

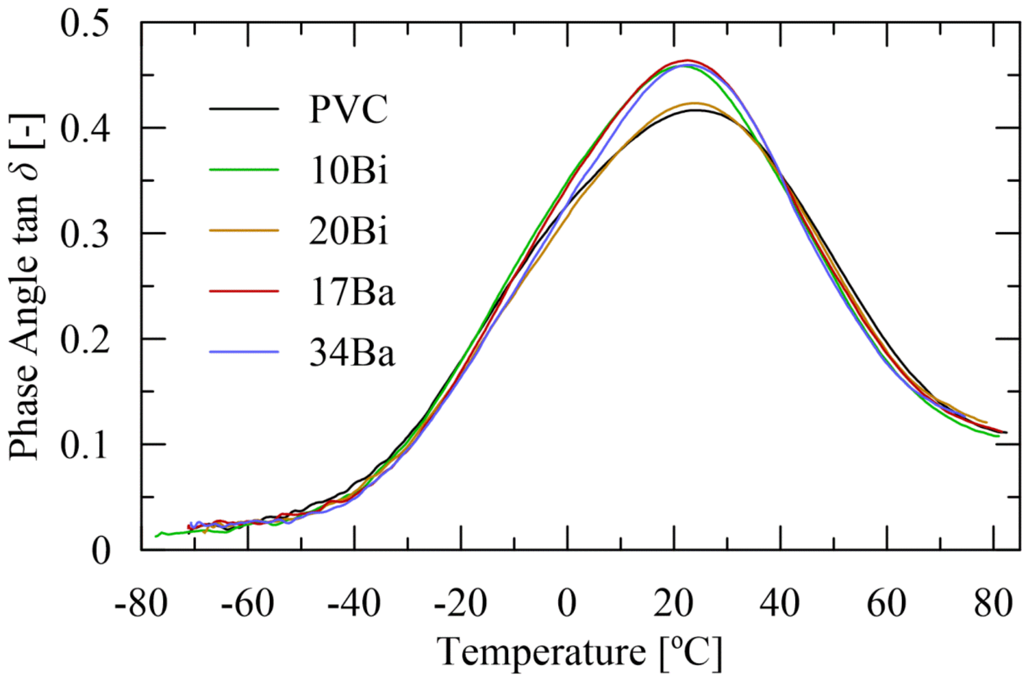

Figure S5. A comparison of phase angles tan δ of pure PVC and all four compounds.
